# Confounding by gender and academic year masks null effects of a Cooperative Training Community framework on undergraduate research outcomes: a mixed-methods study

**DOI:** 10.1186/s12909-026-09194-8

**Published:** 2026-04-16

**Authors:** Yongxiang Yuan, Anji Ren, Kai Huang, Shuang Zhao, Xiang Chen

**Affiliations:** 1 Department of Dermatology, Xiangya Hospital, Central South University, Changsha, Hunan China; 2 Xiangya School of Medicine, Central South University, Changsha, Hunan China; 3 Furong Laboratory, Changsha, Hunan China; 4 Hunan Key Laboratory of Skin Cancer and Psoriasis, Xiangya Hospital, Central South University, Changsha, Hunan China; 5 National Engineering Research Center of Personalized Diagnostic and Therapeutic Technology, Changsha, Hunan China; 6 Stomatological Center, Xiangya Hospital, Central South University, Changsha, Hunan China; 7 National Clinical Research Center for Geriatric Disorders, Xiangya Hospital, Central South University, Changsha, Hunan China

**Keywords:** Undergraduate medical education, Tiered mentorship, Selection bias, Self-efficacy, Confounding factors, Research productivity

## Abstract

**Purpose:**

To evaluate the Cooperative Training Community (CTC) framework for medical undergraduate research training using a mixed-methods design, and to examine the extent to which observed group differences in research outcomes are attributable to the CTC intervention versus confounding by gender and academic year.

**Method:**

This retrospective mixed-methods study was conducted at Xiangya School of Medicine, Central South University, China using data collected in April 2022. Qualitative data comprised transcripts from a semi-structured focus group with one CTC team. Quantitative data were drawn from surveys completed by 295 undergraduates (64 CTC, 231 non-CTC) and 207 graduates (17 CTC, 190 non-CTC), assessing research engagement, self-efficacy, creative efficacy, and innovation performance. Unadjusted group comparisons were followed by multivariate logistic regression adjusting for gender and academic year. A pre-specified sensitivity analysis was conducted in the sophomore subgroup (*n* = 88), the only academic year stratum in which gender distribution did not differ significantly between CTC and non-CTC students (*p* = 0.129), allowing a more controlled within-stratum comparison.

**Results:**

Unadjusted analyses suggested that CTC undergraduates outperformed non-CTC peers on multiple research engagement and productivity outcomes. However, CTC and non-CTC groups differed significantly in both gender distribution (female: 73.4% vs. 53.2%, *p* = 0.004) and academic year (*p* < 0.001). After multivariate adjustment, academic year emerged as the most consistent independent predictor of research productivity across all five outcomes examined (all *p* ≤ 0.019). Female gender independently predicted paper publication (OR = 2.503, *p* = 0.009) and award attainment (OR = 2.411, *p* = 0.003). CTC group membership was not an independent predictor of any productivity outcome after adjustment, with the exception of project participation—a structural feature of CTC membership rather than an educational outcome. In the sophomore sensitivity analysis, no significant CTC-related differences were found in any behavioural or productivity measure; however, CTC students reported significantly lower research self-efficacy (mean difference: −17.81, *p* = 0.005, d = 0.639) and creative efficacy (*p* = 0.046, d = 0.449), with the largest deficits in items measuring autonomous higher-order skills. Qualitative findings were uniformly positive, consistent with social desirability effects in a non-anonymous focus group setting. Among graduate students, the small CTC subsample (*n* = 17) precluded definitive conclusions; however, a gender-stratified sensitivity analysis found that CTC graduates reported stronger perceptions of research-thesis disconnection than non-CTC peers in both male and female subgroups (Δ ≈ 0.45–0.52), suggesting role strain independent of the group’s gender imbalance.

**Conclusion:**

In this single-institution study, the apparent advantages of CTC participation were largely attributable to confounding by gender and academic year rather than the framework’s educational effects. Academic year was the strongest independent predictor of research productivity, underscoring the primacy of accumulated research exposure over training model. CTC participation was associated with lower self-efficacy for autonomous research skills among year-matched undergraduates, consistent with theoretical predictions regarding over-scaffolding. These findings highlight the methodological importance of adjusting for demographic confounders in educational intervention evaluations, and suggest that future iterations of structured collaborative training frameworks should incorporate deliberate mechanisms to foster learner autonomy alongside participation.

**Supplementary Information:**

The online version contains supplementary material available at 10.1186/s12909-026-09194-8.

## Background

Undergraduate engagement in research is an essential component of modern medical education. Beyond clinical competencies, research training cultivates critical thinking, scientific awareness, and proficiency in evidence-based practice, which are positively associated with student’s learning outcomes [1–3]. Historically, undergraduates have contributed to landmark discoveries, such as Charles Best’s role in insulin purification, demonstrating their latent potential [4].

Globally, medical schools integrate research into undergraduate curricula using diverse approaches [5]. In high-income countries, structured programs have yielded significant benefits: 83.9% of students in the USA and Canada engage in faculty-mentored research through integrated tracks or summer programs, resulting in higher publication rates and academic career pursuit [2]. European approaches include mandatory full-time research projects in the Netherlands and doctoral thesis requirements in Germany, though completion rates remain low without early methodological training [6]. In contrast, students in low- and middle-income countries face systemic barriers such as inadequate mentorship and infrastructure, resulting in limited research participation [7–9].

The traditional model of research training is a tutor-graduate structure, where undergraduates are seldom involved [1, 10, 11]. Although undergraduates possess willingness to engage in research, their participation is hindered by limited resources, insufficient opportunities, experience, and unfamiliarity with ongoing projects [12–14]. Crucially, as recent studies from neighboring countries and broader Asian contexts have highlighted, these challenges are not unique to our institution; they represent shared regional barriers where time constraints and a lack of structured mentorship severely limit early research exposure [15–17]. Within this structure, even when undergraduates join laboratories, they are often assigned repetitive or menial tasks, preventing them from acquiring substantial knowledge or understanding [18]. Simultaneously, this model burdens all participants: graduate students are burdened with basic operational work that slows overall research progress ; tutors struggle to effectively mentor undergraduates due to time constraints, missing opportunities to identify future talent and maintain laboratory productivity [19].

To address these challenges, a Cooperative Training Community (CTC) framework was developed at our institution. integrating undergraduates, graduates, and tutors into a hierarchical yet collaborative research ecosystem. Initial unadjusted comparisons suggested that CTC participation was associated with higher research engagement and productivity among both undergraduates and graduates. However, as with many single-institution educational interventions evaluated without randomisation, the CTC and non-CTC groups differed substantially in baseline demographic characteristics—most notably in gender distribution and academic year composition—raising the possibility that observed outcome differences reflected pre-existing group differences rather than the effects of the framework itself.

This study therefore pursues two related aims. The primary aim is to rigorously evaluate the independent effect of CTC participation on undergraduate and graduate research outcomes after adjustment for gender and academic year as potential confounders. The secondary aim is to examine whether CTC participation is associated with differences in research self-efficacy and creative efficacy—outcomes not captured by behavioural or productivity measures alone—using a demographic sensitivity analysis in a year-matched subgroup. By explicitly addressing confounding and attending to both objective and subjective outcome dimensions, this study seeks to contribute not only to the evidence base on collaborative research training models, but also to the methodological discourse on how educational interventions should be evaluated in non-randomised settings.

## Method

### The CTC framework implementation

**Fig. 1:**
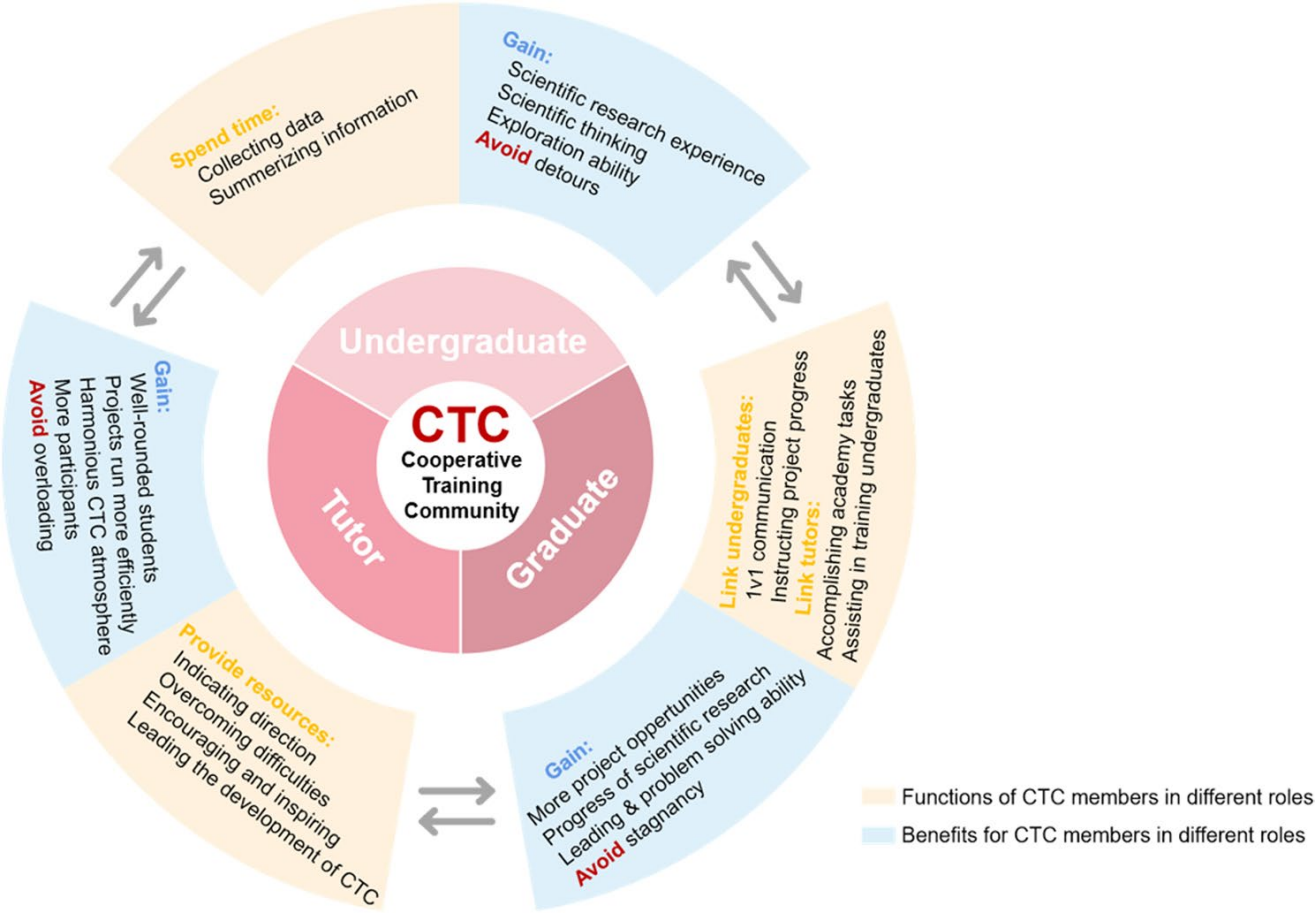
The Composition and significance of the CTC framework

The CTC is a collaborative instructional framework that integrates undergraduates, graduates, and tutors into a synergistic training ecosystem (Fig. 1). The CTC model delineates role-specific functions and mutual benefits within a structured hierarchy. Undergraduates gain research experience by undertaking progressive responsibilities (e.g., data collection, literature summarization, and basic analytical tasks). Graduate students serve as intermediary mentors, providing project oversight and targeted training. Tutors provide strategic guidance, resource allocation, and developmental leadership. This structured interaction aims to create a cyclical growth mechanism to develop undergraduate competencies, enhance graduate mentoring skills, and allow tutors to focus on strategic guidance.

### Study design and setting

This study employed a retrospective, mixed-methods design, analyzing data collected at a medical school in China in April 2022. The study population comprised undergraduate and graduate students majoring in clinical medicine, stratified into CTC and non-CTC groups (undergraduates and graduates). At the time of data collection, all participants provided verbal informed consent. For the current study, all data were fully anonymized prior to analysis. Ethical approval for this retrospective analysis was obtained from the Medical Ethics Committee of our institution (Approval No.2025101767).

### Qualitative method and design

To gain an in-depth understanding of participant perceptions of the CTC model, this study analysed transcripts from a semi-structured focus group conducted in April 2022 with one CTC team, comprising one tutor, two graduate students, and five undergraduates. As this study employed a retrospective design, the qualitative data represent part of the original 2022 data collection, in which a single CTC team was purposively selected to provide initial process-level insight into the framework’s perceived functioning. Participants received refreshments but no monetary incentives.

The 120-minute session was facilitated by a trained moderator and a note-taker, audio-recorded, and fully transcribed. A predetermined set of open-ended questions explored role functions, perceived benefits, and relational dynamics within the CTC structure.

Qualitative data were analysed using reflexive thematic analysis following the six-phase framework described by Braun and Clarke [20]: familiarisation with data, generating initial codes, searching for themes, reviewing themes, defining and naming themes, and producing the report. Two researchers, both involved in the study design but neither serving as tutors nor students within the CTC programme, independently coded the transcripts using NVivo (version 11.0; QSR International). Discrepancies were resolved through discussion until consensus was reached. Methodological triangulation between the two coders was used to enhance credibility. Thematic saturation was considered reached when no new codes emerged from the data within the single focus group session; we acknowledge that this represents a pragmatic rather than formal application of saturation given the single-session design.

We note that the qualitative component carries inherent limitations. The use of a single CTC team restricts the transferability of findings, and the non-anonymous group setting may have introduced social desirability effects, potentially inflating the positivity of reported experiences. These limitations are discussed further in the context of the quantitative findings.

### Quantitative method and design

Quantitative data were collected in April 2022 from two separate questionnaires (distributed via WJS.CN).

The undergraduate questionnaire captured (1)Demographic characteristics; (2)Research engagement profile; (3)Innovation Efficacy Scale [21]; (4) Research Self-efficacy Scale [22]; and (5) Knowledge Innovation Performance Scale [23] (see Supplementary Material S2).

The graduate questionnaire captured (1) Demographic characteristics; (2)Mentoring practices; (3)Research Engagement Scale [24]; (4) Knowledge Innovation Performance Scale; and (5)Tutor-graduate Relationship Questionnaire [24] (see Supplementary Material S3).

Quantitative analyses were performed using SPSS version 19.0. Internal consistency was examined using Cronbach’s α and McDonald’s ω. Descriptive statistics were computed for all variables. For unadjusted comparisons, independent samples t-tests were used for normally distributed continuous variables and chi-square tests for categorical variables. Statistical significance was set at *p* < 0.05. Effect sizes were reported as Cohen’s d for continuous outcomes and odds ratios (OR) with 95% confidence intervals for binary outcomes.

To examine the independent contribution of CTC participation after accounting for potential confounders, multivariate binary logistic regression models were constructed for each binary outcome of interest, simultaneously entering CTC group membership, gender, and academic year (treated as a categorical variable with five levels) as predictors. Ordinal outcomes were analysed using ordinal logistic regression with the same covariate structure. All multivariate analyses were conducted in the full undergraduate sample (*n* = 295). Graduate multivariate analyses were not performed due to the prohibitively small CTC subgroup size (*n* = 17), which would have resulted in severe model overfitting given the number of covariates relative to the minimum cell frequencies observed.

### Sensitivity analysis in the sophomore stratum

Prior to conducting subgroup analyses, we examined gender distribution across academic year strata within the undergraduate sample to identify a stratum in which demographic comparability between CTC and non-CTC students could be reasonably assumed. The sophomore cohort (Year 2; CTC *n* = 32, non-CTC *n* = 56) was the only stratum in which gender distribution did not differ significantly between groups (female: 75.0% vs. 58.9%, *p* = 0.129), and in which academic performance ranking was similarly balanced (*p* = 0.891). Within this stratum, independent samples t-tests were used to compare research self-efficacy and creative efficacy scores between CTC and non-CTC students, without further covariate adjustment, as the primary demographic confounders were judged to be adequately balanced by design. This analysis was conducted post-hoc in response to the multivariate finding that CTC group membership did not independently predict productivity outcomes, in order to examine whether CTC participation was nonetheless associated with differences in subjective competence beliefs.

### Graduate sensitivity analysis

Given the gender imbalance between CTC and non-CTC graduate groups (male: 70.6% vs. 27.4%, *p* < 0.001) and the infeasibility of multivariate adjustment, a descriptive gender-stratified comparison was conducted for the item assessing perceived disconnection between research activities and thesis work—the graduate outcome showing the most consistent signal across unadjusted analyses. Mean scores were compared within male and female strata separately to assess whether the observed pattern was directionally consistent independent of the group gender imbalance.

## Results

### Qualitative findings

The undergraduates had been involved in the CTC group for periods ranging from six months to four years. Among the graduates, one was a doctoral student and the other a master’s student. The following major themes emerged from thematic analysis of the focus group transcript:

#### Complementary demand 

Motivations for joining a CTC team reflected complementary needs among all three participant groups. Undergraduates sought systematic training and research exposure unavailable through formal curricula. Graduate students, burdened by clinical duties and repetitive low-complexity tasks, valued the operational support provided by undergraduates. Tutors prioritised building a sustainable talent pipeline.

#### Graduate students as intermediary communicators 

Graduate students were identified as pivotal intermediaries between tutors and undergraduates. Their relative proximity in age and skill level enabled more approachable and constructive mentorship than direct tutor-undergraduate interaction. As one undergraduate noted:

“When communicating directly with the tutor, the gap in ability feels overwhelming. I often worry that I won’t understand what the tutor says, and I feel afraid to speak up.”

#### Task-embedded training through role differentiation

Graduate students provided direct, task-embedded training to undergraduates, progressing from simple to complex responsibilities. Compared with tutors, graduates were perceived as more patient and available. One undergraduate contrasted this with other experiences:

“In other research training programs, graduate students only assigned us tasks from the tutor. We submitted the work, but because the tutor was too busy, we rarely got any feedback. I couldn’t even tell whether I had done it well or not.”

#### Perceived efficiency gains through task distribution 

The distribution of tasks across three participant levels was perceived to reduce redundancy and improve overall research output. Participants recommended an optimal ratio of one graduate mentor per two to five undergraduates.

#### Dynamic role progression over time

 Roles within CTC teams were described as dynamic over time, with undergraduates progressing from observers to active participants and eventually independent executors within three to six months. Nearly all participants expressed willingness to continue participating.

Qualitative findings were uniformly positive. The interpretive implications of this pattern are discussed in relation to the quantitative findings below.

### Quantitative findings: undergraduates

A total of 295 undergraduates and 207 graduates participated in the quantitative surveys. All multi-item scales demonstrated good to strong internal consistency (Cronbach’s α range: 0.768–0.980).

#### Baseline characteristics (Table 1)

Among the 295 undergraduate participants, CTC students (*n* = 64) differed significantly from non-CTC students (*n* = 231) in gender distribution (female: 73.4% vs. 53.2%, χ²=8.367, *p* = 0.004) and academic year composition (*p* < 0.001), with CTC students disproportionately concentrated in Year 2 (50.0% vs. 24.2%). Academic performance ranking did not differ significantly between groups (*p* = 0.329), indicating comparable baseline academic ability. Given these imbalances, gender and academic year were treated as covariates in all subsequent inferential analyses.

**Table 1:** Baseline characteristics of undergraduate participants by CTC group membership (*n* = 295)

Variable	Category	CTC (*n* = 64)	Non-CTC (*n* = 231)	Total (*n* = 295)	χ²	*p*
Gender	Female	47 (73.4)	123 (53.2)	170 (57.6)	8.367	0.004**
	Male	17 (26.6)	108 (46.8)	125 (42.4)		
Academic year	Year 1	1 (1.6)	59 (25.5)	60 (20.3)	31.093	< 0.001**
	Year 2	32 (50.0)	56 (24.2)	88 (29.8)		
	Year 3	8 (12.5)	58 (25.1)	66 (22.4)		
	Year 4	16 (25.0)	42 (18.2)	58 (19.7)		
	Year 5	7 (10.9)	16 (6.9)	23 (7.8)		
Academic performance ranking	Top 25%	27 (42.2)	74 (32.0)	101 (34.2)	3.434	0.329
	25–50%	20 (31.3)	73 (31.6)	93 (31.5)		
	50–75%	13 (20.3)	56 (24.2)	69 (23.4)		
	Bottom 25%	4 (6.3)	28 (12.1)	32 (10.8)		
Regular research engagement	Yes	37 (57.8)	101 (43.7)	138 (46.8)	3.996	0.046*
	No	27 (42.2)	130 (56.3)	157 (53.2)		
Direct graduate mentorship received	Yes	33 (51.6)	79 (34.2)	112 (38.0)	6.415	0.011*
	No	31 (48.4)	152 (65.8)	183 (62.0)		

Data presented as n (%) unless otherwise stated

*CTC* Cooperative Training Community

* *p* < 0.05 ** *p* < 0.01

#### Unadjusted comparisons

Before covariate adjustment, CTC undergraduates showed higher rates of regular research engagement (57.8% vs. 43.7%, *p* = 0.046), more frequent direct mentoring by graduate students (51.6% vs. 34.2%, *p* = 0.011), higher rates of project participation (78.1% vs. 58.0%, *p* = 0.009), and higher-level award attainment (*p* = 0.020) compared to non-CTC peers. Given the significant group differences in gender and academic year, these unadjusted comparisons should be interpreted with caution.

#### Multivariate analyses

Results of the multivariate binary logistic regression models are presented in Table 2. Academic year emerged as the most consistent independent predictor across all five outcomes examined, reaching statistical significance in every model (all *p* ≤ 0.019), with higher academic year associated with greater research productivity across the board. Female gender independently predicted paper publication (OR = 2.503, 95% CI: 1.257–4.985, *p* = 0.009) and award attainment (OR = 2.411, 95% CI: 1.345–4.321, *p* = 0.003). CTC group membership was independently associated with project participation (OR = 2.382, 95% CI: 1.222–4.644, *p* = 0.011); however, as noted in the Methods, project participation reflects a structural feature of CTC membership rather than an independently acquired educational outcome. For the remaining four outcomes—paper publication, patent application, award attainment, and written works—CTC group membership was not an independent predictor after adjustment (all *p* ≥ 0.055).

**Table 2:** Multivariate logistic regression: independent predictors of research productivity outcomes among undergraduate students (*n* = 295)

Outcome	Predictor	OR	95% CI	*p* value
Paper publication	CTC membership	2.021	0.986–4.142	0.055
	Academic year	3.086	2.226–4.278	< 0.001**
	Gender (female)	2.503	1.257–4.985	0.009*
Patent application	CTC membership	1.102	0.560–2.167	0.778
	Academic year	1.772	1.349–2.328	< 0.001**
	gender (female)	1.358	0.752–2.449	0.310
Award attainment	CTC membership	1.663	0.871–3.174	0.123
	Academic year	2.836	2.103–3.826	< 0.001**
	gender (female)	2.411	1.345–4.321	0.003*
Project participation †	CTC membership	2.382	1.222–4.644	0.011*
	Academic year	1.626	1.237–2.139	< 0.001**
	Gender (female)	1.498	0.903–2.486	0.117
Written works	CTC membership	1.431	0.651–3.146	0.373
	Academic year	1.467	1.066–2.018	0.019*
	Gender (female)	0.821	0.408–1.650	0.579

Academic year modelled as a categorical variable (Year 1–5). Gender coded as female = 1, male = 0. * *p* < 0.05 ** *p* < 0.01

*OR* Odds ratio, *CI* Confidence interval

† Project participation reflects a structural feature of CTC membership (CTC students are embedded within ongoing research projects by design) and should not be interpreted as an independent educational outcome of the framework

#### Sophomore sensitivity analysis (Table 3)

In the sophomore subgroup (CTC *n* = 32, non-CTC *n* = 56), no significant between-group differences were found in any behavioural or productivity measure, including regular research engagement (*p* = 0.715), project participation (*p* = 0.360), paper publication (*p* = 0.800), patent application (*p* = 0.956), award attainment (*p* = 0.306), or written works (*p* = 0.814). In contrast, CTC sophomores reported significantly lower research self-efficacy (mean: 109.94 ± 28.01 vs. 127.75 ± 27.79, mean difference: −17.81, 95% CI: −30.09 to − 5.54, *p* = 0.005, d = 0.639) and creative efficacy (total score: 26.53 ± 5.09 vs. 28.73 ± 4.78, *p* = 0.046, d = 0.449) than their non-CTC peers. Item-level analysis identified the largest efficacy deficits in domains requiring autonomous higher-order judgment: ensuring data reliability (*p* < 0.001, d = 0.809), formulating research questions (*p* = 0.003, d = 0.679), presenting research results (*p* = 0.004, d = 0.658), working independently within a research team (*p* = 0.004, d = 0.648), and selecting appropriate research designs (*p* = 0.011, d = 0.580). In contrast, no significant differences were observed for structured participation items such as literature retrieval (*p* = 0.119) or adherence to academic ethics (*p* = 0.639).

**Table 3:** Sophomore sensitivity analysis: research self-efficacy and creative efficacy outcomes among Year-2 undergraduates (CTC *n* = 32 vs. non-CTC *n* = 56)

Domain	Item	CTC (*n* = 32) Mean ± SD	Non-CTC (*n* = 56) Mean ± SD	Mean difference (95% CI)	*p*	d
Research self-efficacy	Total score	109.94 ± 28.01	127.75 ± 27.79	−17.81 (− 30.09 to − 5.54)	0.005**	0.639
	Formulating research questions	5.31 ± 1.80	6.50 ± 1.72	−1.19 (− 1.96 to − 0.42)	0.003**	0.679
	Ensuring data reliability	5.47 ± 1.92	6.82 ± 1.51	−1.35 (− 2.09 to − 0.62)	< 0.001**	0.809
	Selecting appropriate research design	5.69 ± 1.84	6.64 ± 1.53	−0.95 (− 1.71 to − 0.20)	0.011*	0.580
	Selecting data analysis method	5.47 ± 1.87	6.54 ± 1.89	−1.07 (− 1.90 to − 0.24)	0.012*	0.568
	Presenting research results orally	5.41 ± 1.86	6.66 ± 1.93	−1.25 (− 2.09 to − 0.42)	0.004**	0.658
	Visualising research findings	5.59 ± 1.78	6.84 ± 1.81	−1.25 (− 2.04 to − 0.46)	0.002**	0.694
	Working independently in a research team	5.72 ± 1.89	6.86 ± 1.68	−1.14 (− 1.91 to − 0.37)	0.004**	0.648
	Discussing research ideas with others	6.13 ± 1.70	6.91 ± 1.82	−0.79 (− 1.57 to − 0.00)	0.049*	0.442
	Writing clearly and logically	6.22 ± 1.75	7.05 ± 1.35	−0.83 (− 1.50 to − 0.16)	0.015*	0.553
	Interpreting research conclusions	5.88 ± 1.62	6.86 ± 2.03	−0.98 (− 1.82 to − 0.15)	0.022*	0.519
	Writing a publishable manuscript	4.84 ± 2.62	6.05 ± 2.23	−1.21 (− 2.26 to − 0.16)	0.024*	0.509
Creative efficacy	Total score	26.53 ± 5.09	28.73 ± 4.78	−2.20 (− 4.36 to − 0.04)	0.046*	0.449
	Creatively achieving self-set goals	3.13 ± 0.79	3.57 ± 0.85	−0.45 (− 0.81 to − 0.08)	0.017*	0.538
	Creative effort leading to success (most situations)	3.34 ± 0.87	3.73 ± 0.75	−0.39 (− 0.74 to − 0.04)	0.030*	0.489
	Creatively overcoming difficulties and challenges	3.34 ± 0.79	3.71 ± 0.73	−0.37 (− 0.70 to − 0.04)	0.029*	0.493

Only items with statistically significant between-group differences are presented. Total scale scores are also included. Mean difference = CTC minus non-CTC. d = Cohen’s d

* *p* < 0.05 ** *p* < 0.01

### Quantitative findings: graduates

#### Baseline characteristics (Table 4)

Among the 207 graduate participants, the CTC group (*n* = 17) differed markedly from the non-CTC group (*n* = 190) in gender distribution (male: 70.6% vs. 27.4%, *p* < 0.001)—notably in the opposite direction to the undergraduate sample. Academic year distribution did not differ significantly between groups (*p* = 0.154). Given the small CTC subgroup size, all graduate analyses should be considered exploratory.

**Table 4:** Baseline characteristics and selected unadjusted outcomes among graduate participants (*n* = 207)

Variable	Category	CTC (*n* = 17)	Non-CTC (*n* = 190)	Total (*n* = 207)	Statistic	*p*
Gender	Female	5 (29.4)	138 (72.6)	143 (69.1)	13.647	< 0.001**
	Male	12 (70.6)	52 (27.4)	64 (30.9)		
Degree type	Academic master’s	9 (52.9)	78 (41.1)	87 (42.0)	0.974	0.808
	Professional master’s	4 (23.5)	51 (26.8)	55 (26.6)		
	Academic doctoral	2 (11.8)	33 (17.4)	35 (16.9)		
	Professional doctoral	2 (11.8)	28 (14.7)	30 (14.5)		
Academic year	Year 1	1 (5.9)	54 (28.4)	55 (26.6)	5.261	0.154
	Year 2	9 (52.9)	59 (31.1)	68 (32.9)		
	Year 3	6 (35.3)	66 (34.7)	72 (34.8)		
	Year 4	1 (5.9)	11 (5.8)	12 (5.8)		
Direct undergraduate mentorship assigned	Yes	7 (41.2)	21 (11.1)	28 (13.5)	12.105	0.001**
	No	10 (58.8)	169 (88.9)	179 (86.5)		
Patent applications (n, mean ± SD)	—	2.18 ± 2.21	0.34 ± 0.99	—	t = − 3.385	0.004**
Written works (n, mean ± SD)	—	1.29 ± 1.83	0.28 ± 0.74	—	t = − 2.260	0.038*
Award attainment	Yes	12 (70.6)	37 (19.5)	49 (23.7)	22.564	< 0.001**
	No	5 (29.4)	153 (80.5)	158 (76.3)		
Research-thesis disconnection (mean ± SD)	—	2.41 ± 1.12	1.81 ± 0.94	—	t = − 2.485	0.014*

Data presented as n (%) or mean ± SD as appropriate. All graduate analyses are exploratory given the small CTC subgroup (*n* = 17)

* *p* < 0.05 ** *p* < 0.01

#### Unadjusted comparisons

Unadjusted analyses showed that CTC graduates were significantly more likely to be assigned to provide direct guidance to undergraduates (41.2% vs. 11.1%, *p* = 0.001). CTC graduates reported higher overall research engagement, driven by behavioural engagement (*p* = 0.005) and active engagement (*p* = 0.006) subscales. Innovation performance outcomes were substantially higher in the CTC group across multiple domains, including patent applications (*p* < 0.001), written works (*p* = 0.001), project participation (*p* = 0.007), and award attainment (*p* < 0.001). CTC graduates also reported stronger perceptions that their research activities were unrelated to their thesis work (mean: 2.41 ± 1.12 vs. 1.81 ± 0.94, *p* = 0.014). Multivariate adjustment was not conducted due to model instability resulting from the small CTC subgroup size (*n* = 17) relative to the number of covariates required.

#### Graduate sensitivity analysis (Table 5)

To assess whether the observed difference in perceived research-thesis disconnection was attributable to the marked gender imbalance between groups, a descriptive gender-stratified comparison was conducted. CTC graduates reported higher perceived disconnection than non-CTC peers in both the male subgroup (CTC: 2.50 vs. non-CTC: 1.98) and the female subgroup (CTC: 2.20 vs. non-CTC: 1.75), with comparable effect magnitudes across strata (Δ ≈ 0.45–0.52). This directional consistency across gender strata suggests that the observed association is unlikely to be solely attributable to the group gender imbalance.

**Table 5:** gender-stratified sensitivity analysis: perceived research-thesis disconnection among graduate participants

Subgroup	CTC mean	Non-CTC mean	Δ (CTC − Non-CTC)	*n* (CTC / Non-CTC)
Male subgroup	2.50	1.98	+ 0.52	12 / 52
Female subgroup	2.20	1.75	+ 0.45	5 / 138

Item wording: ‘My research activities are unrelated to my thesis work’ (5-point Likert scale; higher scores indicate stronger perceived disconnection). Δ = CTC mean minus non-CTC mean. Formal statistical testing was not conducted due to small cell sizes

## Discussion

### The illusion of efficacy: confounding by demographic and academic variables

The primary finding of this study is that the unadjusted associations between CTC participation and improved undergraduate research outcomes were almost entirely attenuated after adjustment for gender and academic year, with no consistent independent effect of the CTC framework observed across four of the five core productivity outcomes. This represents a textbook example of Simpson’s Paradox in educational research [25]: the aggregated association between CTC participation and positive outcomes was driven entirely by severe baseline demographic imbalances between groups, rather than a true educational effect of the framework itself. This critical finding challenges the uncritical advocacy of the CTC framework in prior descriptive evaluations, shifting the analytical focus from unadjusted programmatic praise to a rigorous examination of how confounding variables can distort perceptions of educational intervention effectiveness.

Academic year emerged as the strongest and most consistent independent predictor of research productivity across all outcomes, with higher year associated with significantly greater odds of all research outputs. This finding aligns with the well-established “time-on-task” effect in educational research [26]: accumulated duration of research exposure, rather than the hierarchical structure of the training model, is the primary driver of undergraduate research productivity. Critically, the only remaining nominally significant association for the CTC group—“project participation”—must be interpreted not as an independently acquired competency, but as a structural prerequisite of CTC membership (as all CTC undergraduates are embedded in ongoing projects by default). This underscores a key limitation of unadjusted evaluations: the CTC framework functions not as a de novo catalyst for research capability, but as a demographic filter that disproportionately aggregates pre-existing talent, creating the illusion of educational effectiveness.

### Gender as an independent predictor of research outcomes

Female gender was a robust independent predictor of paper publication and competition award attainment, even after adjustment for CTC participation and academic year. This novel finding provides critical nuance to the evolving regional literature. A recent nationwide survey of 3,423 Chinese medical undergraduates confirmed that demographic factors significantly dictate research attitudes, with male students generally reporting higher baseline research interest and initial participation [27]. However, our multivariate analysis reveals a striking divergence: while broad initial participation may lean male nationally, female gender independently drives the most competitive, advanced outputs (papers and awards) in our cohort. In the Chinese context, female medical students make up a significant majority of undergraduate enrollments yet face persistent structural barriers to academic career advancement, including underrepresentation in leadership and implicit gender biases in specialist promotion [28]. The higher proactive research productivity observed in our female participants likely reflects a strategic response to these systemic inequities. Female students may utilize high-level research outputs as a critical mechanism to build hyper-competitive profiles for postgraduate admission, compensating for structural disadvantages rather than reflecting a simple generalized difference in research interest.

### The self-efficacy penalty and the risk of over-scaffolding

The most striking secondary finding was that, in the only demographic stratum with balanced gender distribution (sophomore year), CTC participation was associated with significantly lower research self-efficacy and creative efficacy, with no corresponding benefits in behavioral or productivity outcomes. Critically, the efficacy deficits were concentrated in autonomous higher-order research skills (e.g., formulating research questions, designing independent protocols, interpreting complex data), with no differences observed for basic structured tasks (e.g., literature retrieval, adherence to academic ethics).

This pattern aligns directly with educational theory on scaffolding and over-scaffolding. The classic scaffolding framework posits that structured guidance from more experienced peers reduces initial barriers to learning for novice students, but must be gradually faded as competence develops to foster autonomous problem-solving [29]. When scaffolding remains static and overly structured, as in the CTC model’s fixed hierarchical task allocation, it can inadvertently create “learned helplessness” for higher-order skills: students become proficient at completing assigned tasks, but do not develop confidence in designing and executing research independently [30]. Our finding that CTC sophomores reported lower self-efficacy for autonomous skills, despite equivalent research exposure duration to non-CTC peers, provides empirical support for this theoretical risk in structured collaborative training models. Furthermore, this psychological cost aligns with recent investigations into Chinese medical undergraduates, which reveal that intensive research training often significantly elevates student stress levels and psychological burden when the pedagogical structure focuses heavily on operational output rather than holistic competency development [31]. The fixed hierarchical execution in the CTC framework exacerbates this exact vulnerability, extracting labor without granting the cognitive autonomy necessary to build true self-efficacy.

### Methodological discordance and social desirability bias

The uniformly positive qualitative findings from the focus group stand in stark contrast to the null effects and self-efficacy deficits observed in the anonymous quantitative survey. This discrepancy is most likely explained by social desirability bias inherent to the non-anonymous focus group setting: all participants were active, ongoing members of a single CTC team, with their tutor present in the session [32]. This environment creates strong incentives to report positive experiences and avoid critical feedback, particularly regarding the framework implemented by the attending tutor. We explicitly acknowledge this limitation in our qualitative methods, and interpret the focus group findings as descriptive of perceived benefits among active CTC members, rather than an objective assessment of the framework’s effectiveness.

### Academic fragmentation and mentor role strain

The exploratory graduate cohort findings suggest that CTC participation may be associated with role strain for graduate student mentors. Despite higher unadjusted research engagement and productivity, CTC graduates in both male and female strata reported stronger perceptions that their research activities were unrelated to their thesis work. This finding is independent of the severe gender imbalance in the graduate CTC group, and likely reflects the unintended burden of undergraduate mentorship: graduate students may take on additional research projects outside their thesis scope to create structured tasks for their undergraduate mentees, leading to fragmentation of their own research focus and role strain between their primary thesis responsibilities and their secondary mentoring duties [33].This observation strongly resonates with recent regional studies from neighboring Asian contexts, which emphasize that expanding undergraduate research without adequate structural support places profound logistical and supervisory burdens on intermediary mentors [17].

### Methodological and pedagogical implications

This study has two core implications for the field. Methodologically, it demonstrates the critical risk of overestimating intervention effects in non-randomised educational evaluations without rigorous adjustment for demographic confounders [34]. Most published evaluations of undergraduate research training programs rely exclusively on unadjusted between-group comparisons, which will almost universally overestimate effects in voluntary programs, where more motivated and higher-achieving students are more likely to participate. We strongly recommend that future evaluations incorporate pre-specified confounding adjustment and sensitivity analyses in balanced demographic strata, as we have done here, to validate core findings.

Practically, while the CTC framework’s hierarchical collaborative structure may reduce initial barriers to undergraduate research entry, our findings highlight critical risks that must be addressed in future iterations. First, static, overly structured task allocation must be replaced with a scaffolding fading model: graduate mentors should gradually reduce guidance and increase undergraduate autonomy as skills develop, to foster rather than hinder self-efficacy for higher-order research skills. Second, safeguards must be implemented to mitigate role strain for graduate student mentors, including clear limits on mentoring workload and alignment of undergraduate tasks with the graduate student’s own thesis research, to avoid fragmenting their research focus. Finally, voluntary application-based models will inherently amplify existing inequities in research access; more equitable, curriculum-integrated implementation of the CTC framework would reduce selection bias and ensure equal access for all students, regardless of pre-existing motivation or academic connections.

### Limitations

This study has several important limitations that must be acknowledged when interpreting the findings:

- Causal inference limitations: This is a single-center cross-sectional study, which cannot establish causal relationships between CTC participation and research outcomes. We cannot rule out reverse causality: students with higher pre-existing research motivation, academic ability, or access to mentorship may be more likely to apply for and join CTC teams, rather than CTC participation leading to improved outcomes.
- Qualitative research limitations: The qualitative component is limited to a single focus group with one CTC team, including the team’s tutor, which severely restricts transferability of findings. The non-anonymous group setting introduces significant social desirability bias, which likely explains the uniformly positive results. We only included active, ongoing CTC members, with no input from students who left the program, introducing further selection bias. We reached only pragmatic saturation within the single session, rather than formal theoretical saturation across multiple participants and teams.
- Quantitative research limitations: The graduate CTC sample size is extremely small (*n* = 17), which precludes multivariable adjustment and stable inferential analysis; all graduate cohort findings are purely exploratory and descriptive, and cannot support definitive conclusions. We were unable to adjust for unmeasured confounders, including baseline research experience, individual academic motivation, tutor resource access, and family socioeconomic support, which may contribute to residual confounding. All data were collected via self-report questionnaires, which are vulnerable to social desirability bias, and we did not collect objective administrative data to verify self-reported research outputs. We conducted multiple statistical comparisons across numerous outcomes, which increases the risk of Type I error inflation, despite Bonferroni correction for the 5 primary outcomes.
- External validity limitations: This study was conducted at a single medical school in central China, with only clinical medicine students included. The findings may not be generalizable to other medical-related specialties, other institutional contexts, or other national/regional medical education systems.

## Conclusion

In this single-institution cross-sectional study, the apparent benefits of CTC participation on undergraduate medical student research outcomes were almost entirely attributable to confounding by gender and academic year, rather than independent educational effects of the framework itself. After rigorous statistical adjustment, academic year (reflecting accumulated research exposure) emerged as the strongest and most consistent predictor of research productivity, while female gender independently predicted higher odds of paper publication and competition award attainment. Critically, in the only demographic stratum with balanced gender distribution (sophomore year), CTC participation was associated with significantly lower research self-efficacy and creative efficacy, particularly for autonomous higher-order research skills, consistent with theoretical predictions of over-scaffolding in highly structured collaborative training models. Uniformly positive qualitative findings were most likely explained by social desirability bias in the non-anonymous focus group setting, and were not supported by the anonymous quantitative survey results.

These findings have two core implications for medical education research and practice. Methodologically, this study demonstrates the critical risk of overestimating intervention effects in non-randomised educational evaluations without rigorous adjustment for demographic confounders, and highlights the value of pre-specified sensitivity analyses in balanced demographic strata to validate core findings. Practically, while the CTC framework’s hierarchical collaborative structure may reduce initial barriers to undergraduate research entry, future iterations of this and similar models must incorporate deliberate scaffolding fading mechanisms to foster autonomous research skill development, alongside safeguards to mitigate role strain for graduate student mentors.

## Supplementary Information

Supplementary Material 1.

Supplementary Material 2.

Supplementary Material 3.

## Data Availability

The datasets used and/or analysed during the current study are available from the corresponding author on reasonable request.

## Abbreviations

CTC: Cooperative Training Community
LMICs: low-to-medium-income countries
