# Supplementary material for: Confounding by gender and academic year masks null effects of a Cooperative Training Community framework on undergraduate research outcomes: a mixed-methods study
Source: BMC Med Educ. 2026 Apr 16;26:888. doi: 10.1186/s12909-026-09194-8 (PMC13227753; doi:10.1186/s12909-026-09194-8)
Supplement: Supplementary file 1 — Supplementary Material 1. [file 12909_2026_9194_MOESM1_ESM.docx]

## Supplementary Material S1: Semi-structured Interview Guide

### Focus Group Interview Protocol for CTC Scientific Research Team Study

**Pre-Interview Instructions**

1. Participants were randomly selected (excluding the team leader)
2. No incentives provided beyond refreshments
3. Audio recording obtained with verbal consent
4. Semi-structured format with predetermined open-ended questions and probes
5. Continued until thematic saturation achieved

**Interview Questions**

**1. Role Introduction and Duration**

Please introduce your role within the team (undergraduate student, graduate student, or mentor) and specify the duration of your participation in this CTC project.

**2. Participation Motivation and Needs**

For Undergraduate/Graduate Students:

Why did you choose to participate in this team? What problems did you hope to solve or what needs did you expect to fulfill through this project?

For Mentors:

Why did you decide to organize this type of team? What problems did you aim to solve or what needs did you expect to meet through this project?

**3. Team Model Comparison**

Do you think this CTC model team differs from other mentor-graduate teams or mentor-graduate-undergraduate teams? If so, what are the specific differences?

**4. Benefits and Outcomes**

For Undergraduate/Graduate Students:

Have you gained benefits from participating in this team? If so, what specifically?

For Mentors:

Do you think organizing this team has been beneficial for the collective group, yourself personally, graduate students, and undergraduate students respectively? What are the specific benefits?

**5. Role Definition and Justification**

Please describe the roles that mentors, graduate students, and undergraduate students play within the team (leader, facilitator, participant, observer, etc.). What is the basis for your assessment?

**6. Relational Patterns and Evidence**

Please describe the interpersonal relationship patterns within the team (leader-supervisor-supervisee, collaborators, etc.). What evidence supports your judgment?

**7. Most Memorable Experience**

Please describe the most memorable or impactful experience you have had while participating in this team.

How did this experience influence your perspective on research or collaboration?

Did this experience change how you interact with other team members?

**8. Future Participation Intentions - Undergraduates**

For Undergraduate Students:

When you advance to the graduate level, would you actively apply for or promote the establishment of a CTC team and take on the corresponding graduate student role? Why or why not?

**9. Future Participation Intentions - Graduates**

For Graduate Students:

When you become a mentor, would you actively promote the establishment of CTC teams and assume the mentoring role? Why or why not?

**10. Model Evaluation: Strengths and Weaknesses**

What do you consider to be the advantages of the CTC model? What do you see as its disadvantages or limitations?

**11. Recommendations for Improvement**

In what aspects do you think the CTC model could be improved in future implementations?

**Post-Interview Protocol**

Thank participants for their insights

Confirm confidentiality agreements

Provide researcher contact information

Schedule follow-up if needed for member checking

**Methodological Note:** Two authors trained in qualitative methodology independently coded focus group transcripts for themes. Each researcher defined codes and assigned quotations to categories, then met to reconcile differences and refine coding categories and themes. Consensus was required from the entire research team for identifying final themes. Triangulation of data from multiple analysts and perspectives contributed to the credibility and dependability of qualitative findings.
