# Supplementary material for: Confounding by gender and academic year masks null effects of a Cooperative Training Community framework on undergraduate research outcomes: a mixed-methods study
Source: BMC Med Educ. 2026 Apr 16;26:888. doi: 10.1186/s12909-026-09194-8 (PMC13227753; doi:10.1186/s12909-026-09194-8)
Supplement: Supplementary file 2 — Supplementary Material 2. [file 12909_2026_9194_MOESM2_ESM.docx]

**Undergraduate participation in research willingness and situation survey**

**Part I Demographic characteristics**

1. Your date of birth is [fill in the blank] *

_________________________________

2. Your gender [Multiple choice] *

| ○ man |
| --- |
| ○ woman |

3. Your grade [Multiple choice] *

| ○ First year of university |
| --- |
| ○ sophomore year of college |
| ○ Third year of university |
| ○ Fourth year of undergraduate study |
| ○ 5th year of undergraduate study |

4. What is your academic ranking in the same grade and major since you entered the university [Multiple choice] *

| ○ Top 25% |
| --- |
| ○ Top 25 to 50 percent |
| ○ Top 50 to 75 percent |
| ○ 25% after 25 |

**Part II: Research engagement profile**

5. Do you carry out scientific research activities regularly (e.g., participate in projects, write papers, attend group meetings, etc.) [Multiple choice] *

| ○ yes |
| --- |
| ○ deny |

6. How long ago you first started your scientific research [Multiple choice] *

| ○ Less than a year |
| --- |
| ○ 1-2 years |
| ○ 2-3 years |
| ○ 3 years or more |

7. The cumulative duration of your scientific research activities is about [Multiple choice] *

| ○ Less than a year |
| --- |
| ○ 1-2 years |
| ○ 2-3 years |
| ○ 3 years or more |

8. How many hours on average do you spend on research activities per week [fill in the blank] *

_________________________________

9. When your research supervisor is guiding your research activities, does he/she often assign graduate students to give you direct guidance [Multiple choice] *

| ○ Yes (please jump to question 10) |
| --- |
| ○ No (please skip to question 11) |

10. What do you think is the essence of your relationship with the graduate student? [Multiple choice] *

| ○ co-worker |
| --- |
| ○ Mentor-mentee |

11. If you include a graduate student in your research activities for a long-term, stable direct guidance, what do you expect the relationship with the graduate student to be? [Multiple choice] *

| ○ co-worker |
| --- |
| ○ Mentor-mentee |

12. What role do you want to play in your scientific research activities? [Multiple choice] *

| ○ Leaders (responsible for the direction of scientific research tasks, guidance at key nodes, etc.) |
| --- |
| ○ Leader (responsible for the progress, arrangement and implementation of scientific research tasks, and coordinating with other roles) |
| ○ Participants (complete part of the group research tasks according to the division of labor and arrangement) |
| ○ Bystanders (who mainly study and observe, with little involvement in scientific research tasks) |

13. In fact, what role do you think you usually play in scientific research activities? [Multiple choice] *

| ○ Leaders (responsible for the direction of scientific research tasks, guidance at key nodes, etc.) |
| --- |
| ○ Leader (responsible for the progress, arrangement and implementation of scientific research tasks, and coordinating with other roles) |
| ○ Participants (complete part of the group research tasks according to the division of labor and arrangement) |
| ○ Bystanders (who mainly study and observe, with little involvement in scientific research tasks) |

**Part 3: Innovative efficacy scale**

14. Please evaluate according to your own actual situation. [Matrix single choice] *

|  | 1. Completely disagree | 2. Disagrees | 3. Can't tell | 4. Agreement | 5. Full agreement |
| --- | --- | --- | --- | --- | --- |
| I can creatively accomplish most of the goals I set for myself. | ○ | ○ | ○ | ○ | ○ |
| When faced with difficult tasks, I am sure that I can do them creatively. | ○ | ○ | ○ | ○ | ○ |
| In general, I can get meaningful results in creative ways. | ○ | ○ | ○ | ○ | ○ |
| I am sure that in most cases my creative efforts will succeed. | ○ | ○ | ○ | ○ | ○ |
| I am confident that I can creatively overcome difficulties and challenges. | ○ | ○ | ○ | ○ | ○ |
| I am confident that I can creatively complete a variety of tasks. | ○ | ○ | ○ | ○ | ○ |
| I can accomplish most tasks creatively compared to others. | ○ | ○ | ○ | ○ | ○ |
| Even when the task is difficult, I can show creativity. | ○ | ○ | ○ | ○ | ○ |

**Part IV. Research Self-efficacy Scale**

15. Please evaluate your confidence in your ability to complete the following tasks based on your actual situation. [Multiple choice] *

|  | 0 (no confidence) | １ | ２ | ３ | ４ | ５ | ６ | ７ | ８ | 9 (very confident) |
| --- | --- | --- | --- | --- | --- | --- | --- | --- | --- | --- |
| Follow academic ethics. | ○ | ○ | ○ | ○ | ○ | ○ | ○ | ○ | ○ | ○ |
| Conduct a literature search. | ○ | ○ | ○ | ○ | ○ | ○ | ○ | ○ | ○ | ○ |
| After reading the relevant literature, I had a lot of research ideas. | ○ | ○ | ○ | ○ | ○ | ○ | ○ | ○ | ○ | ○ |
| Discuss your research ideas with classmates or teachers. | ○ | ○ | ○ | ○ | ○ | ○ | ○ | ○ | ○ | ○ |
| Conduct a literature review. | ○ | ○ | ○ | ○ | ○ | ○ | ○ | ○ | ○ | ○ |
| Review journal articles from the perspectives of theoretical basis, research design or data analysis methods. | ○ | ○ | ○ | ○ | ○ | ○ | ○ | ○ | ○ | ○ |
| Ask questions that can be studied. | ○ | ○ | ○ | ○ | ○ | ○ | ○ | ○ | ○ | ○ |
| Present your research ideas to the teacher or group in oral or written form. | ○ | ○ | ○ | ○ | ○ | ○ | ○ | ○ | ○ | ○ |
| The writing is concise and logical. | ○ | ○ | ○ | ○ | ○ | ○ | ○ | ○ | ○ | ○ |
| Conduct appropriate research designs. | ○ | ○ | ○ | ○ | ○ | ○ | ○ | ○ | ○ | ○ |
| Measure the variables under study. | ○ | ○ | ○ | ○ | ○ | ○ | ○ | ○ | ○ | ○ |
| The collected data is guaranteed to be reliable through trial testing, expert evaluation and other methods. | ○ | ○ | ○ | ○ | ○ | ○ | ○ | ○ | ○ | ○ |
| Choose the right data analysis method. | ○ | ○ | ○ | ○ | ○ | ○ | ○ | ○ | ○ | ○ |
| Visualize your research results with pictures or graphics. | ○ | ○ | ○ | ○ | ○ | ○ | ○ | ○ | ○ | ○ |
| Work independently in a research team. | ○ | ○ | ○ | ○ | ○ | ○ | ○ | ○ | ○ | ○ |
| Present your research results orally at academic conferences or in research teams. | ○ | ○ | ○ | ○ | ○ | ○ | ○ | ○ | ○ | ○ |
| Be clear about the shortcomings of your research. | ○ | ○ | ○ | ○ | ○ | ○ | ○ | ○ | ○ | ○ |
| Explain your research findings. | ○ | ○ | ○ | ○ | ○ | ○ | ○ | ○ | ○ | ○ |
| Determine future research directions. | ○ | ○ | ○ | ○ | ○ | ○ | ○ | ○ | ○ | ○ |
| Write a publishable manuscript. | ○ | ○ | ○ | ○ | ○ | ○ | ○ | ○ | ○ | ○ |

**Part V: Knowledge Innovation Performance Scale**

16. Your research output [matrix single choice] *

|  | ０ | １ | ２ | ３ | ４ | ５ | 6 above |
| --- | --- | --- | --- | --- | --- | --- | --- |
| Number of individual and collaborative papers | ○ | ○ | ○ | ○ | ○ | ○ | ○ |
| Number of patents filed by individuals and participating teams | ○ | ○ | ○ | ○ | ○ | ○ | ○ |
| Number of individual and co-authored publications | ○ | ○ | ○ | ○ | ○ | ○ | ○ |
| Number of personal and other projects participated in | ○ | ○ | ○ | ○ | ○ | ○ | ○ |
| Number of awards won by individuals and participating teams in scientific research or competitions | ○ | ○ | ○ | ○ | ○ | ○ | ○ |

17. Level of scientific research output

The highest level of your personal and collaborative paper publication [Multiple choice] *

| ○ not have |
| --- |
| ○ Domestic non-core journals |
| ○ Domestic core journals |
| ○ Domestic CSCD/CSSCI journals |
| ○ International general journal (non-SCI) |
| ○ International SCI journal |
| ○ Special journals |

18. Level of scientific research output

The highest award you have won individually or as a member of a team in scientific research projects or competitions [Multiple choice] *

| ○ not have |
| --- |
| ○ adj. school-level |
| ○ city level |
| ○ provincial level |
| ○ national level |
| ○ international class |
