# Supplementary material for: Confounding by gender and academic year masks null effects of a Cooperative Training Community framework on undergraduate research outcomes: a mixed-methods study
Source: BMC Med Educ. 2026 Apr 16;26:888. doi: 10.1186/s12909-026-09194-8 (PMC13227753; doi:10.1186/s12909-026-09194-8)
Supplement: Supplementary file 3 — Supplementary Material 3. [file 12909_2026_9194_MOESM3_ESM.docx]

**A questionnaire on the influence of different types of research teams on graduate students' scientific research**

**Part I Personal Information**

1. Please enter your date of birth: [Fill in the blank] *

_________________________________

2. Your gender [Multiple choice] *

| ○ man |
| --- |
| ○ woman |

3. The category of degree you are currently pursuing [Single choice] *

| ○ Professional master's degree |
| --- |
| ○ Academic master's students |
| ○ Professional doctoral students |
| ○ Doctoral candidate in academic |

4. Your grade [Fill in the blank] *

_________________________________

**Part II Mentoring practices**

5. Do your supervisors often assign you to give direct guidance to undergraduates when they are conducting research activities [Multiple choice] *

| ○ Yes (please jump to question 6) |
| --- |
| ○ No (please skip to question 7) |

6. What do you think the nature of your relationship with the undergraduate is? [Multiple choice] *

| ○ co-worker |
| --- |
| ○ Mentor-mentee |

7. If your supervisor assigns you to directly supervise an undergraduate student while guiding the undergraduate student in scientific research activities, what is the essence of your relationship with that undergraduate student? [Multiple choice] *

| ○ co-worker |
| --- |
| ○ Mentor-mentee |

8. What role do you want to play in scientific research activities? [Multiple choice] *

| ○ Leader (responsible for the direction of scientific research tasks, guidance at key nodes, etc.) |
| --- |
| ○ Leader (responsible for the progress, arrangement and implementation of scientific research tasks, and coordinating with other roles) |
| ○ Participants (complete part of the group research tasks according to the division of labor and arrangement) |
| ○ Bystanders (who mainly study and observe, with little involvement in scientific research tasks) |

9. In fact, what role do you think you usually play in scientific research activities? [Multiple choice] *

| ○ Leader (responsible for the direction of scientific research tasks, guidance at key nodes, etc.) |
| --- |
| ○ Leader (responsible for the progress, arrangement and implementation of scientific research tasks, and coordinating with other roles) |
| ○ Participants (complete part of the group research tasks according to the division of labor and arrangement) |
| ○ Bystanders (who mainly study and observe, with little involvement in scientific research tasks) |

**Part III: Research Engagement Scale**

10. Please evaluate your confidence in your ability to complete the following tasks based on your actual situation and fill in the corresponding number below. [Multiple choice matrix] *

|  | 1. Not at all | 2. Discrepancy comparison | 3. Basically in line with | 4. Relatively good | 5 fully compliant |
| --- | --- | --- | --- | --- | --- |
| I treat research as a formal job | ○ | ○ | ○ | ○ | ○ |
| The only reason to do research is to get a job | ○ | ○ | ○ | ○ | ○ |
| Research is a part of my life journey | ○ | ○ | ○ | ○ | ○ |
| Scientific research has no social significance | ○ | ○ | ○ | ○ | ○ |
| Scientific research activities are rich and attractive to me | ○ | ○ | ○ | ○ | ○ |
| The scientific research activities I engaged in had nothing to do with writing my thesis | ○ | ○ | ○ | ○ | ○ |
| There is a serious disconnect between theory and practice in scientific research | ○ | ○ | ○ | ○ | ○ |
| I think it is necessary to look for research methods and ideas from other disciplines | ○ | ○ | ○ | ○ | ○ |
| Graduate students are the test products of the graduate training system | ○ | ○ | ○ | ○ | ○ |
| The projects I participated in during my postgraduate study (doctoral degree) were highly academic | ○ | ○ | ○ | ○ | ○ |
| Scientific research is only for the completion of projects | ○ | ○ | ○ | ○ | ○ |
| Discuss with classmates and teachers often | ○ | ○ | ○ | ○ | ○ |
| I actively publish papers | ○ | ○ | ○ | ○ | ○ |
| I often take part in experiments (social practice) | ○ | ○ | ○ | ○ | ○ |
| I actively participate in academic conferences | ○ | ○ | ○ | ○ | ○ |
| I collect and organize literature efficiently | ○ | ○ | ○ | ○ | ○ |
| When I'm involved in scientific research, I forget everything | ○ | ○ | ○ | ○ | ○ |
| I like scientific research and can reasonably deal with the relationship between scientific research, study and life | ○ | ○ | ○ | ○ | ○ |
| I have been involved in scientific research continuously | ○ | ○ | ○ | ○ | ○ |

**Part IV: Research Self-efficacy Scale**

11. Your research output [matrix single choice] *

|  | ０ | １ | ２ | ３ | ４ | ５ | 6 above |
| --- | --- | --- | --- | --- | --- | --- | --- |
| Number of individual and collaborative papers | ○ | ○ | ○ | ○ | ○ | ○ | ○ |
| Number of patents filed by individuals and participating teams | ○ | ○ | ○ | ○ | ○ | ○ | ○ |
| Number of individual and co-authored publications | ○ | ○ | ○ | ○ | ○ | ○ | ○ |
| Number of individual and other projects participated in | ○ | ○ | ○ | ○ | ○ | ○ | ○ |
| Number of awards won by individuals and participating teams in scientific research or competitions | ○ | ○ | ○ | ○ | ○ | ○ | ○ |

12. Level of scientific research output

The highest level of your personal and collaborative paper publication [Multiple choice] *

| ○ not have |
| --- |
| ○ Domestic non-core journals |
| ○ Domestic core journals |
| ○ Domestic CSCD/CSSCI journals |
| ○ International general journal (non-SCI) |
| ○ International SCI journal |
| ○ Special journals |

13. Level of scientific research output

The highest award you have won individually or as a member of a team in scientific research projects or competitions [Multiple choice] *

| ○ not have |
| --- |
| ○ adj. school-level |
| ○ city level |
| ○ provincial level |
| ○ national level |
| ○ international class |

**Part V: Tutor-graduate Relationship Questionnaire**

14. To what extent does the following statement reflect how strictly (controlled) the supervisor manages students during interactions with you? Please move the slider to select [Matrix Text Question] [Enter a number from 0 to 10] *

|  |  |
| --- | --- |
| (１) He/she was confident when discussing my paper | ________________________ |
| (２) We always seem to be experts when we do research | ________________________ |
| (３) Give me a lot of advice | ________________________ |
| (４) said I wasn't honest | ________________________ |
| (５) He was very upset with me | ________________________ |
| (６) Dissatisfied with my progress | ________________________ |
| (７) I think I can't be relied on | ________________________ |
| (８) Always hesitant about my proposals | ________________________ |
| (９) I lack training | ________________________ |
| (10) He was very critical of our progress | ________________________ |
| (11) Criticizing my work | ________________________ |

15. To what extent does the following statement reflect the level of trust between you and your supervisor in the process of communication? Please choose [matrix single choice] *

|  | Not at all compatible | Not comparable | accord with | A good fit | Very fit |
| --- | --- | --- | --- | --- | --- |
| The mentor had enough time to talk to me | ○ | ○ | ○ | ○ | ○ |
| The mentor and I meet enough times each month | ○ | ○ | ○ | ○ | ○ |
| Play a major role in the research process of the supervisor's topic | ○ | ○ | ○ | ○ | ○ |
| My supervisor often gave me financial support during my graduate and doctoral studies | ○ | ○ | ○ | ○ | ○ |
| My supervisor recommended me to my peers | ○ | ○ | ○ | ○ | ○ |
| I admire the academic character of my tutor | ○ | ○ | ○ | ○ | ○ |
| I like my supervisor's research ideas | ○ | ○ | ○ | ○ | ○ |
| My supervisor's research methods fit well with me | ○ | ○ | ○ | ○ | ○ |
| I admire my supervisor's ability to manage research projects | ○ | ○ | ○ | ○ | ○ |
| I think mentors are good at handling relationships with colleagues | ○ | ○ | ○ | ○ | ○ |
| I think the tutor is good at dealing with other students | ○ | ○ | ○ | ○ | ○ |
